# Effective Recruitment or Bot Attack? The Challenge of Internet-Based Research Surveys and Recommendations to Reduce Risk and Improve Robustness

**DOI:** 10.2196/60548

**Published:** 2025-03-14

**Authors:** Liesje Donkin, Nathan Henry, Amy Kercher, Mangor Pedersen, Holly Wilson, Amy Hai Yan Chan

**Affiliations:** 1 Department of Psychology and Neuroscience Auckland University of Technology Auckland New Zealand; 2 School of Psychological Sciences University of Western Australia Perth Australia; 3 School of Pharmacy University of Auckland Auckland New Zealand

**Keywords:** internet-based research, research methodology, surveys, data integrity, bot attacks, technology, data manipulation, spam, false, falsification, fraudulent, fraud, bots, research methods, data collection, verify, verification, participants

## Abstract

Internet-based research has exploded in popularity in recent years, enabling researchers to offer both investigations and interventions to broader participant populations than ever before. However, challenges associated with internet-based research have also increased—notably, difficulties verifying participant data and deliberate data manipulation by bot and spam responses. This study presents a viewpoint based on 2 case studies where internet-based research was affected by bot and spam attacks. We aim to share the learnings from these experiences with recommendations for future research practice that may reduce the likelihood or impact of future attacks. The screening and verification processes used are presented and discussed, including the limitations of these. Based on our experience, security and screening within internet-based research platforms are partly effective, but no solution is available to protect researchers completely against bot attacks. Implications for future research and advice for health researchers are discussed.

## Introduction

### Overview

The internet and digital technologies have irretrievably changed the conduct of research. Whether it be participant engagement, digital intervention delivery, data collection, or distribution of findings, technology has allowed researchers to engage in wider-reaching recruitment. Technology has also enabled people to access and participate in research that may not have been previously possible. In particular, health researchers have embraced internet-based recruitment to invite participation from varied population groups easily and to collect data from large samples.

There has been a significant increase in sample sizes in the last decades, intending to increase statistical power, replicability, and generalizability of research findings [1]. The adoption of open science practices and the emergence of global collaborative efforts, such as the Human Connectome Project [2] and the UK Biobank study [3], including tens of thousands of participants, has facilitated the pooling of resources and increased the overall sample sizes in research studies. However, with the increase in public accessibility of internet-based studies comes a potential increase in poor quality or false participants, ranging from careless respondents who respond with insufficient effort [4] to malicious responses such as automated attacks in the form of computer-programmed bots [5,6].

Bots can be fully automated to target internet-based research via automated malware or malicious algorithms [7], or they may be “hybrid” where there is an element of human control. An example of a hybrid bot is when the human may complete the initial survey screening questionnaires and then allow an automated bot or algorithm to complete the remainder of the survey. Bot attacks have existed since the early 2000s and are becoming increasingly common. This increase may be linked to the rise of internet-based paid research panels and crowdsource platforms such as Amazon Mechanical Turk, which bots can leverage. Whether the bots are automated malware or human respondents who are completing surveys for financial gain without meeting study inclusion criteria [8], it is irrefutable that caution must be exercised when conducting an internet-based or remote study [9,10].

Bot attacks must be identified as they impact the integrity of datasets [5,11,12], creating a situation where ineffective interventions may appear effective, and conversely, effective interventions may seem ineffective. Similarly, one of the significant risks of undetected bot attacks is the potential to misrepresent populations [5,13] and influence decision-making based on erroneous data. Misrepresentation is particularly problematic for vulnerable [14] and at-risk populations such as Indigenous communities, who are often poorly serviced by many existing interventions [15], or populations where new effective interventions are desperately needed [13,16]. Thus, misrepresentations of bots as members of this population could have dire consequences.

This paper discusses the viewpoints of strategies researchers can use to help reduce the impact of bots on research. Like several recent papers [5,13,17-21], the researchers were impacted by bot attacks on 2 independent projects hosted on different platforms at 2 institutions. We present case studies (CS1 and CS2) of the 2 research projects (see Multimedia Appendix 1) that were affected by bot attacks and demonstrate the impact these would have had on the demographics of the final dataset. We share the steps the research teams took to identify potential bot participants, outline strategies to mitigate the bots’ effects and reduce the chances of bot attacks in future projects, and provide recommendations for other researchers.

## Ways to Identify Bot Responses

Several signs in both case studies indicated likely bot attacks—both in terms of researcher review of the dataset and the survey metadata and through software flagging functions. We also attempted to develop an algorithm to predict the likelihood of the participant being a bot to help determine authentic participants from bots. These strategies are discussed further.

### Researcher Strategies to Identify Bots

In both studies, recruitment appeared far more efficient than expected, particularly as there was no targeted advertising and the recruitment methods used were broad. Both studies had high numbers of participant registrations in a brief period, with registrations frequently being close together (often less than minutes apart).

Second, study participant demographics were disproportionate to the sample pool, and populations often hard to recruit were overrepresented. As this was more subtle than the high number of registrations, this may not have been detected until data analysis was underway if unusual recruitment patterns were not present. Overrepresentation was particularly obvious for CS2, where we had planned to purposefully recruit based on ethnicity and monitored to ensure we were successful in this recruitment but had yet to start targeted recruitment. It is noted that although the proportion remained high in the corrected dataset, this was overinflated due to the small sample size and likely would not have remained proportionally high once higher numbers or participants were recruited.

Third, a review of participant email addresses indicated that most suspected bot attacks had emails that followed similar patterns (eg, a combination of letters and numbers followed by the exact email domains) or were nonsensical. While not all email users choose not to use their name in their emails, and the desire to protect against identity theft means that some people do not have emails that make sense, it was the similarity between the email addresses that highlighted the differences between suspected bots and the participants that we believed to be genuine.

Finally, unusual responses in the internet-based surveys also highlighted potential bot responses. Specifically, random answering for survey questions as evidenced by inconsistent or incongruent responses, a lack of answers to qualitative or text responses, and patterned survey responses indicated likely bots.

### Use of Survey Platform Data

In terms of using the functionality of the survey platforms, several additional strategies can be used to determine if a bot attack has occurred. Some survey platforms can flag surveys that are likely bots, so this should be used where possible. Although responses are flagged, researchers still need to review the responses as above to identify atypical patterns of responses. While this requires researchers to review survey responses, the flagging system can potentially reduce the time required to identify bot attacks.

### Potential Statistical Prediction to Identify Bots

#### Overview

For CS2, there were two parts to the study; participants were invited (1) to complete the “research” questionnaire, which consisted of 8 forms, and (2) to use the internet-based intervention component of the study accessed through a second external website using their contact details. By cross-validating the information from the intervention platform with the initial research survey data (N=503), we obtained confirmation of true participants (n=27). However, we could not be sure whether the high number of remaining participants were bots or actual participants who did not continue to the next part of the study, although we assumed they were bots.

Yet this scenario presented us with a unique opportunity to attempt to create a bot detection algorithm to distinguish bots from genuine participants in CS2 better using strategies previously recommended in the literature [18,22]. To achieve this, we assigned suspicion scores on a scale of 0-10 to responses that we evaluated as being potentially anomalous based on the following criteria:

#### Survey Completion Time

We tracked the completion rates, completion times of individual forms within the survey, and the time difference between the initial and final form completion. A weak bimodal distribution appeared for completion times in CS2, allowing us to apply a higher suspicion score to completion times of less than 5 minutes.

#### Email Address Analysis

We scrutinized patterns in email addresses, such as random strings of letters and digits, along with unusual domain names, to identify suspicious email patterns typically associated with bots. Higher suspicion scores were applied to unusual email address combinations.

#### Conflicting Response Analysis

We evaluated responses to specific correlated survey questions to detect inconsistencies that might indicate automated responses. For instance, we sought contradictory answers to 2 questions concerning the frequency of experiencing “not being able to stop or control worrying” and “worrying too much about different things” over the past 2 weeks. Greater negative correlations between paired questions were assigned higher suspicion scores.

To construct the bot detection model, we used the extreme gradient boosting (XGBoost; The XGBoost Contributors) algorithm with a logistic regression base model for binary identification of bots and participants. XGBoost is a scalable tree-boosting system that has shown excellent performance in classification tasks due to its proficiency in managing high-dimensional data and capturing intricate patterns [23,24]. We partitioned the dataset, allocating 80% for model training and 20% for testing model performance. Hyperparameters were fine-tuned through experimentation and cross-validation to attain optimal performance. We used receiver operating characteristic curve results to optimize accuracy, sensitivity, and specificity by adjusting the classification threshold. After optimization, the model achieved an overall accuracy of 0.937, with a sensitivity of 0.967 (reflecting a high rate of bot detection), but a specificity of 0.400 (reflecting a poor rate of actual participant detection), for a classification threshold of 0.25. Hence, the optimized model was heavily biased toward detecting potential bots and unable to identify true participants consistently. The model’s balanced accuracy was moderately low at 0.683, reflecting the challenge of simultaneously identifying true positives and true negatives.

Based on this model, the most important features in order of predictive use were the time difference between the first and last form completions within the baseline survey, the time difference between adjacent forms from “unique” participants, the total completion time of the overall survey, the email suspicion score, and conflicting responses for correlated questions. We also ran univariate logistic regressions for individual features to validate the XGBoost model results. Heavy 0 inflation (ie, high numbers of participants with suspicion scores of 0) and poor discriminability were present in each feature, making it impossible to identify bots accurately even when features were combined.

To perform supervised learning this way, we assumed a binary classification of participants versus bots. In reality, the data were imperfectly labeled. Instead of labeling participants as “not bot” or “bot,” the best we could achieve was labeling them as “not bot” or “maybe bot.” Because the study comprised the internet-based research questions and the internet-based intervention, we used the login and intervention usage data to confirm some true participants (as the intervention website required interaction with diary components and activities, thus confirming a true participant). Nevertheless, there remained a high degree of uncertainty regarding the identity of the remaining participants who only completed the first baseline research questionnaire but did not proceed to log into the intervention. Thus, the bot detector’s accuracy is probably exaggerated.

## Ways to Manage a Bot Attack

Researchers should regularly monitor recruitment and data collection to aid in the early identification of bot attacks. Close monitoring is critical soon after studies are advertised on the web, as the risk of a bot attack, based on these 2 studies, seems to occur soon after study advertisement. While frequent data monitoring will not stop a bot attack, it may reduce the breadth of the attack by being able to intervene early. Researchers can monitor data manually by regularly checking recruitment or by setting up alerts for new enrolments, which will signal if a high volume of enrolments occurs over a short time.

### Close or Pause the Survey

Once a bot attack is detected, it is essential to close or pause the survey immediately. In both case studies, new surveys were created and circulated with additional security measures in place, including location screening features, CAPTCHA coding, the use of fraud scores algorithms, and referral restrictions if recruitment occurs through social media (further strategies are discussed in the section “Ways to counteract a bot attack”). Closing or pausing the survey ensures that no new bot enrolments can occur (although settings may allow incomplete surveys to be completed) and extra security measures can be implemented.

### Seek Institutional Support

After identifying the bot attack, several actions were taken to help the research teams consider what to do next. Actions included notifying the respective ethics committees about the attack and seeking consultation and advice about responding. Despite this surge in internet use for health research, there remains limited guidance from health research ethics committees on managing bot attacks or ensuring the validity of internet-based research studies. The emergence of large-scale digital research and artificial intelligence have presented unique challenges in research ethics and safety. Artificial intelligence (AI) and “big data,” particularly digital data, present unique ethical considerations, which institutional research ethics boards may not be adequately equipped to deal with [25]. For example, the issue of transparency is unique to contemporary AI models, as they are often considered “black boxes” where the user is unaware of how the model has reached its conclusion [26]. Accountability remains a complex issue as it is unclear who should be held responsible when AI is involved [27]. In this context, we believe retaining autonomy and “human control” of digital data is paramount. AI can also blur the concept of free and informed consent, and managing privacy becomes more challenging with AI’s ability to identify individuals even after data deidentification [28]. Data bias is also a concern as AI may not always detect or could potentially generate biased results, including harmful gender and racial biases [29]. It is crucial to continue addressing these ethical challenges to ensure responsible and safe implementation of AI technologies.

In both our case studies, several ethical dilemmas arose. The first dilemma was the issue of reimbursement. As there is often a set budget for participant reimbursement, an ethical dilemma arises when the number of claims exceeds those budgeted. Specifically, in studies where actual enrolments exceed planned enrolments, there could potentially be hundreds of people who cannot be reimbursed if the study protocol outlines reimbursement of all respondents regardless of data quality. The second dilemma was the inability to differentiate between bots and actual participants in a way that could 100% confirm which responses were “real” and which were not. Failure to accurately identify between participants and bots could lead to a risk of reimbursing bots and erroneously excluding reimbursement of actual respondents due to budgeting constraints.

Finally, there were concerns about potential reputational risks for the researchers and their academic institutions if the research team reimbursed “fake” responses or missed payment for “real” participants. The respective university ethical and legal teams were consulted about researcher obligations to compensate based on completed answers, even if suspected bot attacks generated these. The standard advice was that the bot attack was a misrepresentative response, and therefore, there was no obligation to compensate, given that the terms of research participation had not been met.

In CS1, where participants were to be sent vouchers for compensation, careful screening of voucher claims revealed only 4 valid registrations (out of over 1200 claims), which were subsequently paid. Where participant contact details were available in CS2, we gained ethical approval to recontact participants to indicate that there had been an attack on the study. We asked participants to complete their baseline data collection again to obtain their compensation. The recompletion rate was approximately 55% and no complaints were received about this by the researchers or the ethics committee.

### Remove Data

Where there was a high probability of being a bot participant, these data were removed from the dataset, and a record was made of this decision. This decision was based on the above identification parameters. Where it was difficult to tell if the participant was a bot-participant or a legitimate participant, this was noted to allow further scrutiny during the longitudinal follow-up and for reporting in publications as appropriate. It is noted that in both case studies, 2 researchers were involved in each review of data and the decision to remove participants based on being a bot. It was also determined that it was important to do this before data analysis occurred, document all research decisions, and inform the respective ethics committees.

## Ways to Counteract Bot Attacks

### Plan Ahead

We believe that all researchers involved in internet-based research should have a bot-management protocol in their data management plan. This could be based on protocols such as Ballard et al [11] and include the frequency of monitoring enrolments for bot attacks, steps to take when the survey is active if a bot attack is suspected, and a data handling and analysis plan for the study and that includes planning for bot-related and suspicious data. There should be a clear justification for removing data, and the dataset should be reviewed independently by 2 reviewers, including any decision made to remove data. Decisions should be documented, and publications related to the study should disclose the level of data removal related to the bot attack. Similarly, ethics committees who have approved the study should be consulted and informed about bot attacks, given the impact on research integrity.

### Study Advertisements

The researchers in this study and others [9,18,30] believe that studies that are advertised on social media, particularly media where bots are highly active and reach are widespread (such as Twitter or Facebook), are more likely to experience bot attacks. The placement of study advertisements should be specific to the study population, where possible, and highlight the benefits of participating in the research other than compensation. Settings on survey platforms can also limit access to pages via routes other than where they were initially posted; for example, in CS1, settings were changed so that only participants who accessed the survey via the Facebook pages where the links were posted were included in the dataset. In addition, only those who accessed the voucher registration page via the survey could enter their details. Using previously verified distribution lists, such as those associated with professional or patient organizations, may reduce the likelihood of bot attacks as the advertising is more targeted.

### Participant Information Sheets and Consent Forms

Participant information sheets and consent forms should highlight the steps researchers will take if they believe a participant has misrepresented themselves. Currently, participant information sheets often outline the inclusion and exclusion criteria and may ask that the participant indicate that they meet these criteria; however, there is no explicit statement about data management for suspected bot attacks. Upon reviewing the participant information sheet for CS2, the ethics committee felt that the wording and context did sufficiently imply that reimbursement was given only if the questionnaires were completed as part of the intervention study, so this provided adequate grounds for researchers to omit reimbursement to participants who did not complete the questionnaires as part of the fuller study. Nevertheless, an ethics amendment was made where the wording was changed to stipulate that reimbursement more clearly was only given if the questionnaires were completed and the study intervention was used as part of participation.

For future studies, we would recommend the inclusion of text such as the following:

Any data that is thought to be generated by a bot or non-human means, is a duplication beyond what is required by the study, or is misrepresentative may be removed by the research team. If this occurs, study compensation for participation, as outlined in this participant information sheet, will not be offered. If you believe your data has been removed unfairly, you may discuss this with the lead researcher, who may ask you for proof of the legitimacy of your claim. If there are issues with data collection, you may be contacted to complete parts of the research again, which you may choose to decline.

Consent forms should also include reference to this, such as “I understand that if my data is believed to be misrepresented it may be removed from the study and I may not receive compensation for participation.” Before including this in external documents, we recommend consulting with your institutional legal team and ethics committee.

### Prescreening of Participants

Designing a 2-step registration process that requires screening for study eligibility prior to accessing the survey and that includes multifactor authentication for identity validation may be 1 way to counter web-crawler bots. A more thorough strategy could be prescreening participants manually via a phone call, but this increases the time intensity of a study [21] and may also create a hurdle for participation by requiring additional steps to be completed. Further, commercial tools for identity verification from public records may also be used depending on the location of the study, ethics committee approval, and the researcher’s ability to access these. However, these tools rely on up-to-date public records and prevent anonymous participation, which again may create barriers.

### Compensation of Participants

Offering financial compensation for research participation is a common strategy when engaging in health-related research, encouraging a breadth of responses [31] and helping to reduce the impact of self-selection bias [32]. Compensating participants is considered an ethical approach to ensuring widespread health research engagement and facilitates participation by financially disadvantaged participants [33,34]. Compensation also recognizes the time participants have contributed to the research project and the value of their contribution. However, compensation means that research can also attract participants who may not be interested in the outcome of the research and are participating for other reasons.

Advertising compensation in study advertisements may increase the likelihood of studies being targeted by bots [5,6,18]. Likewise, direct compensation for internet-based data collection increases the risk of a bot attack, which may be mitigated by offering a prize draw instead. However, a prize draw may be less effective for recruitment [35], may not fairly compensate participants for their time, and may be less likely to recruit or may greater disadvantage more some individuals [36]. Alternatively, offering compensation contingent on completing specific tasks may reduce the likelihood of fraudulent participation, particularly if compensation payment is only made at the end of the study.

Ethics committees recommend offering compensation relative to the degree of involvement of the participant. For example, a 5-minute survey may only be eligible for a prize draw, whereas a study with an in-person 3-hour visit that requires blood tests and medical scans will warrant a higher compensation. However, there is limited guidance on what compensation is expected for different activities, and research teams arbitrarily determine compensation. Thus, studies where researchers offer a higher level of compensation for less effort (such as a short internet-based survey), might be more appealing to bot attacks. Given this, we would advocate for more explicit guidance on research compensation, particularly for internet-based studies.

Clarifying participant identity to claim compensation may also be another barrier for financially motivated bot attacks. Examples of this could be participants being asked to provide identification at the end of the survey to claim compensation and to ensure the participant is a person or the research teams sending vouchers to a postal address rather than an email address. Another potential way to determine authenticity may be using electronic bank transfers to a confirmed bank account rather than digital vouchers similar to case transfer programs that have been trailed in health research [37]. Alternatively, cryptocurrency could also be effective, as the process of claiming blockchain-based rewards could involve complex cryptographic tasks or multifactor authentication methods that are easy for humans but difficult for bots. Cryptocurrency transactions are also publicly verifiable by default on the blockchain, providing a transparent record of each payment (note that cryptocurrency has not yet achieved sufficiently widespread adoption to enable this method, and its use could potentially be seen as contentious [38,39]). Likewise, the requirement to share personal banking information may be a barrier to participation or could preclude people who do not have the financial fluidity to experiment with or access cryptocurrency, which has often been associated with people with more financial freedom [40]. Thus, the use of cryptocurrency may only be viable in limited situations. These strategies could reduce the potential for bots or hybrid bot attacks to be paid and could prevent participants from claiming multiple compensations for multiple completions of the same survey.

### Enhanced Security Settings

Researchers should familiarize themselves with the security options within their survey platforms to reduce bot attacks. Security options include activating settings to reduce multiple responses from the same participant, bot detection features, and CAPTCHA settings. For CAPTCHA, Qualtrics (Qualtrics International Inc) generates scores for each participant on a 0 to 1 scale based on several variables. Scores less than 0.5 are likely bots. It was noted that in the case studies above, this was not enough to deter bots but was a method of helping to identify potential bots. RelevantID settings also use code to determine if participants are taking the survey multiple times, allowing researchers to remove duplicate participants.

### Crafty Survey Development

Two survey design steps may help make it more difficult for bots to access surveys. The first requires registration with an email address, and then potential participants must validate their email address to log on to the internet-based survey. This registration requires more actions to log in, which makes it harder for less sophisticated bots to access the survey. However, as noted in CS2, bots or hybrid bots may be able to overcome this. Additionally, initial screening or inclusion criteria could be set up as individual questions that are randomized in order, and the correct answers need to be selected for inclusion in the final dataset. An alternative could be using questions that require 2 answers that are linked and require only certain answers to indicate a valid participant. An example could include selecting a country of origin from a drop-down list and then entering a postcode [20] which the research team check to validate if this is a real participant.

Surveys can also include questions that are only likely to be completed by bots, not participants. These are called “honey pot” questions, and each survey platform has a different way of creating them. For example, a field may be created that is invisible to humans, but can be detected by a bot. It is also noted that creating these questions in some coding software may be easier for a bot to detect, and recent internet-based threads have recommended that JavaScript (Mozilla Foundation) be the preferred coding platform for creating these fields.

The inclusion of text-based questions that need answering may also help with the identification of bot participants. Examples of this could include a series of questions requiring a typed answer about the country’s capital, what year it is, the city the person lives in, and basic mathematical equations such as “What is one plus three?” Question format can also increase the chance of detecting a bot by directing participants only to select a specific number of items from a list where all items could be answered.

Kay and Saucier [41] have created an internet-based database of 660 items that can be used to detect careless or insufficient-effort responders in survey data. The authors propose that the best way to identify participants who respond in a misleading or invalid way is to add frequency and infrequency items. Frequency items should be endorsed almost by every respondent (eg, it should be illegal to kill an innocent person), and infrequency item statements should be endorsed by almost no respondents (eg, when someone tells a funny joke, I feel angry) [41]. Thus, failure to endorse a frequency item or an infrequency item would likely indicate a bot.

The final recommendation is randomization of survey items or questions that make it difficult for bots to “predict” the question and answer “appropriately,” thus making it easier to identify nonsensical surveys. Similarly, including free text or open text questions may also help to identify bots versus actual participants, as bots are less likely to complete these meaningfully or may replicate answers. Thus, reviewing qualitative answers for quality or content duplication may help identify a bot. Additionally, including qualitative answers may make bot responding more challenging and provide more insights for future research not captured in closed-text answers [14]. It is noted that these strategies individually will not necessarily prevent hybrid people-bot attacks but will increase the difficulty of survey completion. If multiple strategies are used, it will likely be more deterring for the bot and make it easier to identify bot-responding.

### Metadata

The use of IP address tracking may be helpful to identify actual participants compared to bots. Coupled with location filters, IP address tracking can help determine when multiple attempts are made of the same survey and when the participant is not within the geographical catchment of the survey. However, it is noted that virtual private networks can circumnavigate this, and IP address tracking alone is not foolproof. Some ethics committees consider IP addresses to be identifiable information that must be disclosed during the informed consent process if collected. Using this strategy should be approved by the overseeing ethics committee. For further details on using IP addresses to mitigate bots’ effects, see White and Brodhead [8].

Asking participants to provide information that is matched to their IP address or metadata could also be another strategy to validate real participants. This could include strategies such as asking what country the person is in and matching it to an IP address or geolocation or to confirm the time where they are and match it to survey functions which capture the times of both the participant’s browser and the server of the host institution to detect discrepancies [20].

## Challenges

### Overview

There is likely no foolproof way to detect bots and determined bot users could overcome many of these strategies through a human answering some of the screening questions and then running the bot script. Thus, bot-attacks in internet-based research are an ongoing problem to manage. There are also several ethical issues when identifying potential bots.

### Decreased Anonymity

Requiring participants to provide a form of identification prevents researchers from collecting anonymous responses. This may be particularly problematic for studies where participants may be reluctant to participate due to fear, stigma, or where the behavior being studied is illegal [42]. There are strategies to reduce the impact of the potential to be identified, such as the software randomly allocating codes that are not tied to answers or a researcher who does not have access to the survey data (or even the topic) being the contact person for voucher claims. Researchers working in areas where stigma and shame are common may be reluctant to design surveys where participants may need to be identified; however, there is research showing that larger incentives and greater privacy do not necessarily equate to disclosure of more sensitive data [43] and greater anonymity may impact the accuracy of internet-based surveys [44]. Thus, protecting participant identity through deidentification may be the preferred route to prevent bots and increase survey accuracy.

### Removal of Data

Removing bot data is important for research integrity, but this should be done cautiously so as not to remove data belonging to valid participants. This is particularly valid when considering removing data with incomplete answers (particularly qualitative answers), as this may result in removing data from people who may struggle with literacy but also misrepresent attrition and engagement—both critical issues in digital interventions [45-47].

Data removal should be done by 2 independent, blinded researchers and disclosed in publications to ensure transparency and rigor and decrease biases. Similarly, sharing the processes around managing bot-suspected material will aid the research community in developing a consistent approach to bot-data management. Specifically, we believe that researchers should report the methods used to determine bot data, the percentage of data removed due to the data being bot-generated, and the percentage of any suspicious remaining data. As such, we need consistent community guidelines for handling and reporting bot-related data and research impact.

### Abusive Responses or Researcher Protection

Soon after the early closure of one of the studies, the researchers began to receive requests for participation compensation. When “participants” were told that there would be no compensation due to suspected fraudulent behavior, researchers began to receive abusive messages. While this is often not at the level of abuse that has recently been disclosed with the rise of the internet [48,49], receiving such abuse is a risk to the well-being of researchers [50,51]. Given this, a plan is needed to determine if and how to respond to such communications and ensure the researcher’s well-being [50,51].

## Discussion

### Principal Findings

This study presents 2 case studies of bot attacks encountered by our research team and the strategies we used to counter them. Moreover, it underscores the critical challenge facing internet-based survey research, which remains highly susceptible to various forms of malicious interference. Unless novel technologies are developed to counter these threats, the challenge of obtaining trustworthy survey data over the internet will likely become more difficult in future years. Our hope is that this paper can serve as a warning, helping to drive the changes needed to protect the integrity of internet-based survey research moving forward.

As technology evolves, so too will the sophistication of bot attacks. As such, bots are here to stay. Therefore, researchers will need multiple, regularly updated strategies to combat and manage bots’ effects, including institutional support and ethics committees knowledgeable in this space. It is likely that health research will need to consider multiple bot prevention strategies, use multimodal recruitment and data collection, and develop clear guidelines to help researchers manage bot-related data in internet-based research.

Authentic participant data are valuable, and therefore, researchers need algorithms and a decisional process to detect bot data. A method that is overly sensitive to bots may remove actual participants unnecessarily, thus reducing the size of the dataset and may result in the study being underpowered. On the other hand, an algorithm or decisional process with low specificity may allow an excessive number of bot data to be included, reducing data quality and increasing study costs through compensation paid to nonauthentic participants. Further, bots are likely to provide either extremely noisy or biased answers that negatively impact data quality. When creating a bot detection algorithm, it is up to the researcher to decide whether they wish to prioritize sensitivity (the ability to detect participants) or specificity (the ability to detect bots).

When attempting to develop an appropriate algorithm to detect bots, we faced severe issues with zero-inflated data where most participants—whether bots or not—registered a suspicion score of 0 for multiple variables. This indicates a lack of information about the methods used to create the bot. It highlights the possibility that the attack was performed by human actors responding manually to each survey, making it seem more authentic. Yet even with perfect knowledge of our artificial bot dataset, any bot detection algorithm trained on this dataset would endure from severe overfitting issues, as the attack strategies used will likely differ from those used for another project. Consequently, our detection algorithm probably would not generalize well to real-world attacks, except for similarly designed REDCap (Research Electronic Data Capture; Vanderbilt University) surveys. To make this even more challenging, each of the survey tools currently available to researchers (such as Qualtrics, REDCap, or SurveyMonkey) have different programming interfaces and database structures that would each require a unique version of the bot detection algorithm that is configured to their setup. Hence, we remain unaware of any tool that provides sufficient accuracy for bot detection across the wide range of currently used survey tools. Detection is made more difficult by imperfect participant labeling. For example, researchers can rarely be completely certain that an email address belongs to a bot, even in the presence of “obvious” signs such as random strings of letters or digits. Genuine participants wishing to preserve their anonymity on the web may create a dummy email address for responding to surveys or use “hide my email address” systems, which can also create unusual email handles. The best way to counter fake email addresses is by screening potential participants beforehand and sending a private, individual survey link to prescreened addresses rather than making the link publicly available [18]. However, this may not be practical for all projects as it can be labor-intensive and as highlighted in CS2, is not foolproof.

### Implications

Health research often engages vulnerable populations, and special care must be taken to protect privacy and ensure appropriate risk management is available where needed—including the detection of bots that may skew findings. One vulnerable group are the Indigenous populations. Health research must be responsive and capture the needs of indigenous populations who often experience poorer health outcomes. In CS1, bot responses identified as Māori (the Indigenous population of New Zealand) made up 30% of the sample, and after bot measures were in place, the percentage identifying as Māori was 10%. In CS2, “bot” responses inflated the participants that identified as Māori to almost 4 times what we expected in recruitment based on the population composition. Had we not identified the bot, we would have been quite confident in the analysis we made relating to Māori participants, given the high number, despite these results being unrelated to Māori at all. Therefore, there is a risk that failure to detect and adapt to bot technology could lead to misinformation and contribute to poorer outcomes for indigenous populations based on misinformed conclusions.

Given this, we encourage researchers to consider whether internet-based surveys are the best way to obtain a representative sample with high data accuracy. Internet-based recruitment may preclude some people, and although we increasingly see people being connected on the web, regular data connection, access to technology, and technology literacy may not yet be everyone’s privilege, including vulnerable communities. This is particularly pertinent for those where the digital world may be at odds with core and cultural values [52] or where surveys may not accurately capture experiences [53]. Instead, the use of multimodal surveys may reach a broader group, including those who may not be aware of internet-based surveys, may be more responsive to the needs of communities, and may be able better to manage the impact of bots [14].

### Recommendations

From our experiences and literature review, it would seem prudent to develop clear guidelines for conducting internet-based research to reduce the risk of bot attacks and increase the robustness of internet-based research. There are currently few guidelines that exist to guide practice in this area. The Association of Internet Researchers released their latest ethical guidelines for internet research in 2019 [54]. The guidelines provide useful information about data management and security, and consider some of the critical issues that we discuss in our paper, including how to protect the researcher where the researcher’s public identity is known; specific ethical topics such as accountability, trust, and transparency which have different considerations for internet-based research; and issues related to the accuracy of data including in-built biases from algorithms used for collection and how to use metadata. The EQUATOR (Enhancing the Quality and Transparency of Health Research) network refers to 3 reporting guidelines relating to digital health research—the CHERRIES (Checklist for Reporting Results of Internet E-Surveys) checklist—a checklist for reporting results of internet e-surveys [55]; the CONSORT (Consolidated Standards of Reporting Trials)-EHEALTH to standardize the reporting of evaluations of web and mobile interventions [56], and the iCHECK-DH (Guidelines and Checklist for the Reporting on Digital Health Implementations) which is a guideline and checklist on how to report digital health implementations [57,58]. However, these EQUATOR guidelines refer more to the reporting of the internet-based study than the conduct and do not refer to bot attacks or management [59]. We advocate for developing standardized guidance for researchers conducting internet-based research that describe key considerations and a standardized approach to ensuring data accuracy and validity. These guidelines should also include recommendations for reporting when bot attacks may have occurred and how researchers should handle data with regard to this. While we recognize that bot attacks are likely to be ever-evolving and are likely to outpace the development of guidelines, we believe it would still be useful for guidelines or discussion documents for researchers for strategies to mitigate or minimize the potential effects of bots. Similarly, these guidelines or discussion documents may include points to help researchers determine if the strategies required to minimize bot attacks and the probability of obtaining valid data outweighs the risk of an attack, thus determining if internet-based health research is viable.

### Future Directions

Bot attacks will likely become more widespread and more difficult for researchers to detect as botware and attack algorithms become more sophisticated. The experiences of our research group and others [5,13,17-19] highlight a critical, growing problem that deserves more focused researcher attention. Nevertheless, internet-based research has many advantages, such as the ability to reach large numbers of participants and the ability to complete the research remotely. Our experiences should not deter researchers from conducting internet-based research studies. Instead, our paper is a call to action to raise awareness and encourage researchers to consider the risks and benefits of internet-based research. We recommend developing guidelines around detecting and managing bot data in internet-based surveys to help raise awareness of these issues, provide guidance around survey design and data management, and encourage transparency in reporting data that bots may have impacted.

Researchers should also consider the recent exponential development of large language models (LLMs) such as ChatGPT (OpenAI) [60]. Unlike manually programmed bots, LLM-assisted bots can interpret and respond to surveys more coherently, making LLM responses more challenging to detect. This also allows attackers to automate responses to qualitative questions, a task previously reliant on human guidance. We believe it is only a matter of time before LLM-assisted bots become sophisticated enough to respond to any survey design. Consequently, it may become impossible to trust any results obtained from public survey links. Therefore, we recommend that researchers begin implementing more effective security strategies if they have not already done so.

A question that arises at this point, and a potential focus for further research, is whether bot attacks are generalizable across different parts of the world, as different countries have varying protocols for paying research participants [61]. Our experience also suggests that all current research platforms may be vulnerable to illegal bot attacks, and the associated responsibility of software developers to ensure the security and privacy of people is paramount. Thus, bot attacks are likely to be a global issue.

### Conclusions

While internet-based research studies increase the ease of participant recruitment and accessibility to a diverse range of respondents, the rise of sophisticated bot programmers and algorithms to automate survey responses risks invalidating internet-based research. Careful planning of internet-based research study designs and incorporating measures to minimize bot responses, such as using a mix of closed, open-ended, and randomized questions, is necessary to protect internet-based studies from bot attacks. However, these measures should be weighed against the risk of inadvertently disqualifying or turning away real, genuine participants. There is an urgent need for standardized practices and guidelines to be developed to provide researchers with clear guidance on safeguarding against bot attacks and actions to take if a bot attack is suspected. As bot attacks are here to stay, this paper aims to raise researchers’ awareness and create a call to action before the problem becomes more widespread and challenging to manage.

## Appendix

Multimedia Appendix 1 Case studies.

## Abbreviations

AI: artificial intelligence
CHERRIES: Checklist for Reporting Results of Internet E-Surveys
CONSORT: Consolidated Standards of Reporting Trials
CS: case study
EQUATOR: Enhancing the Quality and Transparency of Health Research
iCHECK-DH: Guidelines and Checklist for the Reporting on Digital Health Implementations
LLM: large language model
REDCap: Research Electronic Data Capture
XGBoost: extreme gradient boosting
