# Supplementary material for: Effective Recruitment or Bot Attack? The Challenge of Internet-Based Research Surveys and Recommendations to Reduce Risk and Improve Robustness
Source: Interact J Med Res. 2025 Mar 14;14:e60548. doi: 10.2196/60548 (PMC11953592; doi:10.2196/60548)
Supplement: Multimedia Appendix 1 [file ijmr_v14i1e60548_app1.docx]

## Case study one – fraudulent claims of completion

Case study one (CS1) was run through the Qualtrics platform (1) and offered modest compensation (NZD$20.00 gift voucher) to each participant who completed a survey. The research advertisement specified eligibility criteria (country, parents of children in a specified age range, health experiences). Standard Qualtrics security settings, including CAPTCHA (Completely Automated Public Turing test to tell Computers and Humans Apart), were enabled at the beginning of the survey before the participant information and consent forms. At the end of the survey, participants could click an embedded link to be taken to a separate Qualtrics page and register for the gift voucher. Registration for the gift voucher was separated to protect the anonymity of the survey data.

The invitation was shared with 31 Facebook groups with participants located in New Zealand (NZ). Approximately 12 hours after the invitation was shared, several responses (n=55) were noted, disproportionate to expectations for this study recruitment. Qualtrics had flagged some as potential bots. The study’s lead researcher checked the Qualtrics records and found 1100 voucher claims. The survey was suspended (Qualtrics “pause survey” function) immediately, and all study invitations on social media were deleted.

Further responses continued to be registered while the survey was paused. After seven days, 809 responses were initiated, and there had been 1213 registrations for vouchers. It was subsequently determined that the “pause survey function” allowed unfinished surveys to remain open for seven days before automatically closing, so registration was only stopped after this period. University management, ethics committee, and legal counsel were consulted, with the advice to screen all claims carefully and pay only eligible participants, per the participant information sheet provided.

### Impact on dataset

The initial dataset contained 809 responses (see Table 1 for further comparison between the groups). The data and voucher claims were both screened carefully for eligibility. Repeated answers, acquiescence bias, inconsistencies and duplicated text answers were noted. Only four eligible voucher claims were found and paid. Response times varied significantly, with some lodged in nine seconds and others remaining open for many hours. Due to doubts about the integrity of the dataset, it was discarded.

The survey was restarted with enhanced security, including location screening, CAPTCHA coding, Relevant ID, Duplicate Scores, Fraud Scores, and referral restriction (survey only accessible when link clicked from Facebook; voucher page only accessible when link clicked from survey final page). Following the improved security, 1495 responses were recorded in the new survey and 350 voucher claims over a seven-week recruitment period. These were screened according to the criteria above, with blank responses (including those blocked from continuing based on embedded security, *n*=1025) and invalid Qualtrics security scores (*n*=29) excluded first, then manually screened duplicates (*n*=15) and ineligible participants *(n=*18 outside the age range). It was noted that some participants left the survey open for many hours but then completed and submitted their responses; however, almost all responses were completed within 30 minutes, as expected. The final sample excluded those with excessive missing data (>20% of items) and yielded a sample size of 382. Some participants declined to register for a voucher, with the remaining 350 claims all appearing valid.

Notably, the enhanced security effectively protected the voucher claim system using a Qualtrics weblink that only opened when accessed via the final survey page, thus deterring web crawlers (a search engine bot) and preventing direct access via the URL. Survey data supported the improved process of screening out invalid access, and manual screening from there was simpler. However, this was not perfect and still took time – demonstrating the importance of enhanced security and researcher vigilance in ensuring that online data is valid and genuine.

### Cost to the research

Once the attack was noted, two senior researchers spent approximately 4 hours each reviewing the data and attempting to determine which participants were genuine. A further 8 hours were spent liaising with the institution's information technology team, ethics committee, legal team, and biostatistics consultants to determine the impact on the research and decide what to do. As a result of the time taken to do this and the subsequent modifications and relaunch, data collection was delayed by two weeks.

**Table S1.** A comparison of data sets with the suspected bot data was included, and datasets with the suspected bot data were removed.

|  | Case Study One (CS1) | | Case Study Two (CS2) | |
| --- | --- | --- | --- | --- |
| Recruitment aim | 400 participants (parents across NZ) | | 100 participants (50 Māori and 50 non-Māori) | |
| Survey Platform | Qualtrics | | REDCap | |
| Study type | Cross-sectional survey | | Longitudinal cohort study with five data points | |
| Advertising | 31 Facebook Groups for parents based in New Zealand | | Institutional X posting which researchers reposted. | |
| Compensation | NZD$20.00 per participant | | NZD$20.00 per data point completion | |
| Period recruitment was open for | 12 hours | | 24 hours | |
| Initial security measures | CAPTCHA image check  CAPTCHA coding  RelevantID  Compensation registration through separate Qualtrics link, accessed at end of survey (but not blocking direct access) | | Email address for follow-up questionnaires | |
|  |  | |  | |
| Demographics | First bot-attacked survey | Final dataset with bot data removed | Bot included dataset | Final dataset with the bot data removed |
| Sample size | 809 | 382 | 503 | 27 (5.37%) |
| Age | Parent *m =* 35.9 (*SD* = 5.7)  Child *m* = 7.7 (*SD* = 1.9) | Parent *m* = 38.6 (*SD* = 6.7)  Child *m* = 7.8 (*SD* = 1.9) | *m* = 36.16 (SD = 6.78) | *m* = 34.41 (SD = 5.48) |
| Gender | Parent female *n* = 421 (52.0%)  Male *n* = 289 (35.7%)  Non-binary *n* = 8 (1.0%)  Prefer not to say *n* = 11 (1.4%)  Child female *n* = 246 (30.4%)  Male *n* = 464 (57.4%)  Non-binary *n* = 7 (1.0%)  Prefer not to say *n* = 6 (0.7%) | Parent female *n* = 332 (82.6%)  Male *n* = 61 (15.2%)  Non-binary *n* = 4 (1.0%)  Prefer not to say/missing *n* = 5 (1.2%)  Child female *n* = 193 (48.0%)  Male *n* = 205 (51.0%)  Non-binary *n* = 2 (0.5%)  Prefer not to say/missing *n* = 2 (0.5%) |  |  |
| Ethnicity | NZ European *n=* 381 (47%)  Māori *n* = 242 (29.9%)  Pasifika *n* = 73 (9.0%)  Asian *n =* 29 (3.6%)  Other *n* = 9 (4.4%)  Remainder missing | NZ European *n=* 290 (72.1%)  Māori *n* = 43 (10.7%)  Pasifika *n* = 23 (5.7%)  Asian *n* = 18 (4.5%)  Other *n* = 19 (4.7%)  Remainder missing | Māori (n *=* 382, 76.0%)  Non-Māori (n *=* 121, 24.0%) | Māori (n *=* 23, 85.2%)  Non-Māori (n = 4, 14.8%) |
| Location | Participant claims:  Aotearoa New Zealand n=809 (100%)  Qualtrics geolocation data:  Aotearoa New Zealand *n*=38 (63%)  USA *n*=17 (28.3%)  Hong Kong *n*=2 (3.3%)  Japan *n*=1 (1.7%)  Missing *n* =  746 (92.2%) * | Qualtrics screened geolocation: Aotearoa New Zealand n=382 (100%) | Country of Birth  Aotearoa New Zealand (n *=* 471, 93.6%)  Cook Islands (n *=* 4, 0.8%)  Samoa (n *= 6*, 1.2%)  Australia (n = 4, 0.8%)  China (n = 1, 0.2%)  Japan (n = 1, 0.2%)  India (n = 1, 0.2%)  South Africa (n = 3, 0.6%)  Other (n = 12, 2.4%) | Country of Birth  Aotearoa New Zealand (n *= 26*, 96.3%)  Other (n = 1, 3.7%) |
| Compensation required | $24,000 | $7640 | $41,000 | $2,460 |
| Average time spent on the survey per participant | 34.6 minutes  Range: 9 seconds – 1939 minutes | 34.4 minutes  Range: 3 minutes – 1392 minutes | Baseline response time: 9.38 minutes, Range: 33 seconds - 1 hour and 4 minutes, Mode: 1.33 minutes, Median: 7.20 minutes  Average follow-up surveys: 2.42 minutes, Range: 12 seconds – 1.11.26 minutes, Mode: 1.14 minutes, Median: 1.29 minutes | Baseline response time: 7.49 minutes, Range: 2.23 minutes to 48.54 minutes, Mode: 2.23 minutes, Median: 5.49 minutes  Average follow-up surveys: 3.07 minutes, Range: 16 seconds – 44.36 minutes, Mode: 1.07 minutes, Median: 1.51 minutes |
| Completion rate of follow-up questionnaires | n/a | n/a | Baseline: 100%, n = 503  Follow-up 1: 74%, n = 371  Follow-up 2: 83.5%, n = 420  Follow-up 3: 76%, n = 385  Follow-up 4: 73.6%, n = 371 | Baseline: 100%, n = 27  Follow-up 1: 92.6%, n = 25  Follow-up 2: 88.9%, n = 24  Follow-up 3: 88.9%, n = 24  Follow-up 4: 85.2%, n = 23 |

* Qualtrics does not record geolocation for incomplete surveys

***Ethical Considerations***

Ethical approval was granted to the institutional ethics committee for a period of 3 years (Auckland University of Technology Ethics Committee reference 23/78). As the survey was online and anonymous, as outlined in the participant information sheet and the survey, progressing with the survey beyond the participant information sheet was considered to be an indication of consent. The data collected were anonymous, with a separate Qualtrics page linked from the final page of the survey, enabling participants to register for the NZ $20.00 compensation without their contact details being linked with their survey responses. Subsequent ethical amendments resulting from the bot attack were the enhanced security settings outlined above.

## Case study 2 – faked participants

Case study two (CS2) was an online survey that aimed to evaluate the effect of an online intervention on various outcome measures of health and wellbeing. The data were collected and managed using REDCap electronic data capture tools (2, 3). The original survey target was to recruit 100 participants, including 50 Māori participants, who would be compensated NZD$20.00 for each completed survey. The survey was planned to be repeated at five time points over six months so participants could be reimbursed up to a maximum of NZD$100.00 if all five surveys were completed. The study was launched online via the University faculty X account, and within eight hours, there were over 500 responses, of which 76% identified as Māori. The high number of enrolments in a short period and the high percentage of Māori participants disproportionate to the population of Aotearoa (approximately 16% are Māori (4)) without any Māori-centred recruitment raised suspicions that a bot attack may have occurred.

On further review, it appeared that most of these enrolments were not actual respondents. Given that most participants’ email addresses followed a specific format and had the same email provider, the researchers concluded that it was likely a bot attack. The baseline survey was immediately paused to prevent further responses, whilst ‘anti-bot’ measures were taken, including integrating these measures into the follow-up surveys to ameliorate future issues.

The incident was immediately reported to the ethics committee, and an adverse event report was lodged. A meeting with the Chair of the Ethics Committee and the Ethics Integrity Manager resulted in an ethics amendment and an updated questionnaire that included open-ended free text fields that respondents had to complete to provide feedback on the intervention as a bot-detection measure. A decision was also made to re-invite the initial suspected authentic participants and request that they re-complete the updated questionnaires, and then the original dataset would be discarded.

Bot detection strategies resulted in the exclusion of participant emails and responses that were highly likely to be bots (n=386; 76.7%) and then contacting the remaining participants on their registered email addresses, indicating that we had some issues with survey data collection and asking that they re-complete the survey. Of the 117 (23.3%) participants that we were not confident they were bots, 64 (54.7%) completed the baseline survey again. On reviewing these completed answers, it was determined that 23 (35.9%) were further bot answers based on inconsistencies between baseline surveys on demographics, two (3.1%) were likely duplicate bot attacks, 34 (53.1%) were likely actual participants, and seven (10.7%) were duplicates, most likely to be actual participants. Thus, with the removal of duplicate participants, the cleaned dataset only had 27 (5.4%) likely actual participants.

However, upon examining the follow-up survey completion four weeks later, it was found that approximately 70% of what we deemed were ‘bot’ responses also completed the follow-up questionnaires despite the anti-bot measures in place. The ‘bot’ responses appeared to complete most of the follow-up questionnaires, and a similar pattern of completion rates appeared across consecutive ‘bot’ responses. However, it was noted that the bot completion rates were consistent at approximately 80%, showing much less attrition across time points compared to other web-based longitudinal studies (5, 6).

### Impact on dataset

The initial data set contained 503 responses, with the majority reporting Māori ethnicity (76%). Of the 503 responses, 476 were deemed ‘bot’ or invalid, leaving 27 authentic responses. The age and country of birth appeared similar between the whole data set and the authentic dataset. Data collection recommenced with enhanced security measures (including adding a CAPTCHA to the start of the survey) and open-ended questions to detect ‘bot’ responses. Unfortunately, the CAPTCHA proved unsuccessful at deterring bots, suggesting the possibility of a hybrid bot attack. Therefore, we decided to pause the online survey and only participants who signed up for the intervention were included in the dataset rather than anyone who completed the baseline survey.

### Cost to the research

A total of 24 hours of senior academic time was utilised following the identification of the bot attack and the subsequent liaison with ethics committees, redesigning the survey and identifying participants that were probable bots. A research assistant spent approximately 20 hours reviewing the dataset, removing participants that were thought to be bots in consultation with two senior academics, and redeveloping and deploying the questionnaire. The response to the bot attack delayed the survey by six months

***Ethical Considerations***

Ethical approval was granted to the institutional ethics committee for 3 years (Auckland Health Research Ethics Committee reference AH23110). All participants completed an informed consent process that occurred after the participant information sheet and consisted of an online digital consent form. The data collected were identifiable as this longitudinal study required participants to complete a survey at multiple timepoints. However, data analysis used deidentified data. At registration and at each completed survey time point, participants received an NZ $20.00 gift card to compensate participants for their time. This resulted in a maximum compensation of NZ $100.00 if all time points were completed. As a result of the bot attack, ethics amendments were made consisting of the enhanced security features mentioned above and accessing the intervention being considered an inclusion criteria for compensation and inclusion in the final data analysis.

1. Qualtrics. Qualtrics. Provo, Utah, USA: Qualtrics; 2020.

2. Harris PA, Taylor R, Thielke R, Payne J, Gonzalez N, Conde JG. Research electronic data capture (REDCap)—a metadata-driven methodology and workflow process for providing translational research informatics support. Journal of biomedical informatics. 2009;42(2):377-81.

3. Harris PA, Taylor R, Minor BL, Elliott V, Fernandez M, O'Neal L, et al. The REDCap consortium: building an international community of software platform partners. Journal of biomedical informatics. 2019;95:103208.

4. Statistics New Zealand. Ethnic group summaries reveal New Zealand's multicultural make-up 2020 [Available from: <https://www.stats.govt.nz/news/ethnic-group-summaries-reveal-new-zealands-multicultural-make-up>.

5. Meyerowitz-Katz G, Ravi S, Arnolda L, Feng X, Maberly G, Astell-Burt T. Rates of attrition and dropout in app-based interventions for chronic disease: systematic review and meta-analysis. Journal of medical Internet research. 2020;22(9):e20283.

6. Geraghty AW, Torres LD, Leykin Y, Pérez-Stable EJ, Muñoz RF. Understanding attrition from international internet health interventions: a step towards global eHealth. Health promotion international. 2013;28(3):442-52.
